# The Role of Telemedicine in Prehospital Traumatic Hand Injury Evaluation

**DOI:** 10.3390/diagnostics13061165

**Published:** 2023-03-18

**Authors:** Francisco R. Avila, Rickey E. Carter, Christopher J. McLeod, Charles J. Bruce, Gunel Guliyeva, Ricardo A. Torres-Guzman, Karla C. Maita, Olivia A. Ho, Sarvam P. TerKonda, Antonio J. Forte

**Affiliations:** 1 Division of Plastic Surgery, Mayo Clinic, 4500 San Pablo Rd., Jacksonville, FL 32224, USA; franciscorav93@gmail.com (F.R.A.);; 2 Department of Quantitative Health Sciences, Mayo Clinic, 4500 San Pablo Rd., Jacksonville, FL 32224, USA; 3 Department of Cardiovascular Medicine, Mayo Clinic, 4500 San Pablo Rd., Jacksonville, FL 32224, USA; 4 Department of Plastic and Reconstructive Surgery, The Ohio State University Wexner Medical Center, Columbus, OH 43210, USA

**Keywords:** hand injury, finger injury, wounds and injuries, trauma centers, mHealth, eHealth, telehealth, triage

## Abstract

Unnecessary ED visits and transfers to hand clinics raise treatment costs and patient burden at trauma centers. In the present COVID-19 pandemic, needless transfers can increase patients’ risk of viral exposure. Therefore, this review analyzes different aspects of the remote diagnosis and triage of traumatic hand injuries. The most common file was photography, with the most common devices being cell phone cameras. Treatment, triage, diagnosis, cost, and time outcomes were assessed, showing concordance between teleconsultation and face-to-face patient evaluations. We conclude that photography and video consultations are feasible surrogates for ED visits in patients with traumatic hand injuries. These technologies should be leveraged to decrease treatment costs and potentially decrease the time to definitive treatment after initial evaluation.

## 1. Introduction

According to the US Department of Labor and the US Bureau of Labor Statistics, in 2018, there were 286,810 cases of occupational traumatic upper extremity injuries, of which 123,990 were hand injuries (43.2%) [1]. Additionally, data from 2009 from the National Electronic Injury Surveillance System (NEISS) showed there are 3,468,996 upper extremity injuries resulting from consumer products per year in the US alone. Of these, finger lacerations accounted for most injuries [2]. Health care workers unfamiliar with traumatic hand injuries in certain centers may perceive a lesion to require a higher level of care than it needs. For example, prior studies have shown that a hand surgeon did not ultimately see more than 50% of transferred patients, and up to 66% of these patients did not require surgery within 24 h [3]. These results imply that most cases were not urgent and could have been treated in a nonspecialized facility or at home. Furthermore, alternative consultation methods could help avoid patient transfer [3]. In addition to these latest findings, previous studies examined transferred patients requiring plastic surgery assessment for hand and facial injuries and found that up to 74% of these emergency consults were unnecessary, resulting in an extra annual expenditure of USD 4.6 million [4]. Therefore, unnecessary transfers from low- to high-level facilities for traumatic hand injuries have a substantial economic impact on both patients and the health care system.

Not only do unnecessary transfers have an economic impact on the patient and the health care system, but they also contribute to emergency department (ED) overcrowding worldwide. In pandemic settings, where the number of acute patients requiring urgent care may already be high, this problem is exacerbated. Persistent ED overcrowding can result in an increase in medical errors, emergency bed boarding, and ambulance diversion, thereby compromising patient safety overall [5]. Boarding, which refers to holding patients for extended periods in the ED [5], negatively affects patient outcomes and has been observed to be associated with increased risk of in-hospital mortality for stays exceeding 24 h [6]. The search for solutions to these issues has included increasing the bed turnover rate, increasing bed monitoring, and reevaluating patients after lengthy stays [7]. In addition to these solutions, telemedicine initiatives could also be pursued to reduce the number of low-risk patients visiting the ED and redirect those with severe trauma or disease to appropriate facilities [8]. Recent efforts to reduce overcrowding with telemedicine have had positive results by expanding the provider screening hours using remote triage and assisting patient self-triage [9,10], proving the feasibility of these services.

The World Health Organization defines telemedicine as the delivery of medical services using communication technologies to exchange information to improve diagnosis, treatment, research, evaluation, and education [11]. Although the transition to telemedicine in several medical areas was already ongoing, the COVID-19 pandemic sparked the need to use telemedicine as a vital adjunct to provide continuous care in the setting of social distancing recommendations while reducing the risk of disease transmission [12,13]. To relieve facilities from nonurgent cases and in an effort to decrease the transmission rate of SARS-CoV-2, hand surgery associations are already introducing telemedicine as an important step in traumatic hand injury assessment before possible patient admission [14,15].

Several studies have tested the implementation of telemedicine applications in emergency settings for patient triage, finding reductions in ambulance transports and an increased productivity in emergency medical services [16,17,18,19]. These consequences in turn lead to the redistribution of emergency medical services to patients with more severe injuries. Several studies have already evaluated mobile communication in trauma settings with positive results. For example, some messaging systems have already proven effective for radiograph interpretation in orthopedic trauma consultations, with good intraobserver agreement between phone pictures and desktop images [20]. In some cases, specialized apps to evaluate specific traumatic injuries by patients at home have been developed, with positive feedback [21]. Therefore, it is possible that introducing similar technology to the field of prehospital triage for traumatic hand injuries could be beneficial for both patients and providers. In light of the current pandemic burden, added to the already high costs associated with unnecessary patient transfers, the present review aims to describe the studies using telemedicine to remotely evaluate and triage patients with traumatic hand injuries. Summaries of the studies are provided in Table 1. Lastly, the review is organized into sections describing the most relevant outcomes of the reviewed studies.

## 2. Study Outcomes

### 2.1. Treatment Outcomes

Studies have shown that treatment agreement between evaluations before and after teleconsultation is high, reaching levels up to 95% [23,26]. Moreover, even in cases involving amputations, surgeons were able to correctly identify suitability for finger replantation through photography evaluation, leading to successful treatment [22,25]. Hsie et al. [25] found that phone photography was 90% sensitive and 83% specific for identifying digital replantation potential. At the same time, Lam et al. [24] determined that final treatments following photographic evaluation were largely similar to those obtained via face-to-face assessment. These findings suggest that a photo messaging system could be an effective alternative to traditional emergency department (ED) assessment in pandemic situations and would allow medical personnel to access photographs several times, including them in patient records. Ultimately, implementing this system beyond emergency settings would improve access to specialized care in rural or underserved areas where such services may be limited. Previous experience using telemedicine in rural ED has shown a shorter time to see a provider and a shorter length of stay at the first hospital when patients were transferred [36,37]. Additionally, telemedicine showed equivalent clinical effectiveness and appropriate care processes compared to traditional, in-person care in the ED [38]. Therefore, although telemedicine interfaces in rural settings could depend on the severity of the injury [38], its use in hand trauma warrants further study.

### 2.2. Triage Outcomes

Few studies had evaluated the feasibility of telemedicine interventions for patient triage before the COVID-19 pandemic, and these were primarily focused on nursing triage [8,39]. Having said that, the number of studies indexed in the NIH National Library of Medicine using the terms “triage” and “telemedicine” more than quadrupled from 2019 to 2020, reflecting not only an interest but a necessity in pursuing these technologies. Although most of the studies now focus on telemedicine to triage patients with COVID-19 [40], new studies from all medical and surgical areas are exploring the possibility of implementing telemedicine triage for their emergent patients. We believe that “teletriage” is a promising area in telemedicine that requires further study, particularly with regard to traumatic hand injury evaluation.

Teleconsultation and photographic or video examinations can potentially transform the treatment of patients with traumatic hand injuries. By categorizing their severity through photographs, triage can be expedited, and precise therapy can be administered more quickly, as described by Hsie et al. [23]. In their study, the authors categorized patients into different groups. Patients in group 1 had mild injuries that could be managed with conservative treatment (e.g., secondary intention wound healing, primary closure with or without bone shortening); patients in group 2 were those likely requiring a specialist for skin grafting or local flap coverage; and patients in group 3 were those with severe injuries that required microsurgery (e.g., replantation or free flap coverage) [23].

Moreover, Hara et al. [29] showed that collecting images from EMTs could help send patients to more suitable institutions, while Diver et al. [26] indicated that teleconsultation could have prevented up to 25% of ED visits. The AHTTP has had additional success in lowering hospitalizations and surgeries following ED visits, as well as transfers to higher-level hospitals [32,33]. Furthermore, these systems have prevented unnecessary medical evacuations in army members and civilians in remote locations by providing a timely and appropriate evaluation of their injuries [27,28,30]. The analyzed studies provide evidence that photography or video instructions given to patients enable them to be remotely examined at home or redirected instantly if necessary. This would lower the risk of SARS-CoV-2 spreading while eliminating admission requests that are unwarranted and overburden emergency departments.

### 2.3. Diagnosis Outcomes

Studies of remote photographic evaluations for traumatic hand injuries have revealed diagnostic agreement rates between 87% and 95%, comparable to those obtained for treatment agreement. In three separate studies, Hsie et al. [23,25] and Paik et al. [31] observed high agreement between in-person and photographic assessments. Diver et al. [26] also reported a concordance rate of 95%. These findings suggest that telemedicine can be used as a substitute for face-to-face evaluation when it comes to identifying injury characteristics; however, additional research is required to improve the efficacy of this method for severe lesions that may result in reduced extremity function. These results resemble those found in the literature regarding other acute conditions. For example, despite physicians accurately identifying the total body surface area burned via photographic evaluation, determining the correct burn depth was more challenging, and this telemedicine interface’s accuracy was low [41]. These results were observed even though the image quality displayed on tables and smartphones was comparable, and in some cases, superior to that displayed on conventional computer monitors [42]. Therefore, photographic evaluation might only be helpful in specific acute trauma scenarios, limited to injuries that are not limb or life-threatening.

On the other hand, video evaluations have shown better results, as exemplified in a study by Fonseca et al. [43]. In this study, the authors found high agreement between on-site and remote physician diagnosis and management decisions evaluating facial lacerations in the ED [43]. Based on this and other studies, a recent review deemed videoconferencing a potential tool for facial injury triage [44]. Whether these findings could be translated to hand injury evaluation is uncertain. Although this review includes studies using photographic and video assessment, these focused mainly on using photography. Therefore, such a statement asserting the best telemedicine interface cannot be made at the moment. Further research comparing diagnostic accuracy using photography and video evaluations of traumatic hand injuries is warranted to identify the best remote evaluation method.

### 2.4. Cost Analysis Outcomes

The only study that analyzed cost differences between in-person consultations and remote teleconsultation was that by Tripod et al. [32], who analyzed changes in patient transport costs before and after the implementation of the AHTTP. The authors found a significant reduction in the percentage of costs covered directly by the patient when discharged home, directly from the ED (from 38.5% before to 24.1% after; *p* < 0.001). Therefore, by reducing the number of unnecessary transfers, the program also reduced the cost of patient transport after discharge.

A review performed by de la Torre-Díez [45] found that, despite the majority of cost analyses favoring telemedicine to reduce costs in different medical specialties, not all agreed. A subsequent analysis performed by Eze, Mateus, and Cravo Oliveira Hashiguchi [46] identified that, although telemedicine can be cost-effective in many cases, this assertion cannot be generalized due to poor quality and reporting standards. However, when stratifying by the telemedicine intervention’s purpose, Snoswell et al. [47] reported that it could reduce costs whenever health-system-funded travel and the need for expensive procedural or specialist follow-up were prevented. Thus, although this latter statement supports the findings of this review, it is crucial to consider that these stem from a single study, and further research should be conducted to identify the reduction in costs for total health care costs.

### 2.5. Time Outcomes

Addressing overcrowding in the ED by decreasing waiting and treatment times is a long-standing problem that had already been studied before the start of the current pandemic [48]. One of the most notable examples is that of the Lisa Perry Emergency Center at New York Presbyterian Weill Cornell Medical Center, where low-risk patients were offered a telemedicine visit with an off-site physician [49]. This approach led to patients concluding their ED visits in less than 30 min with high satisfaction rates [49]. In the current review, response time outcomes showed contrasting results, since it was seen by Paik et al. [31] that evaluation by an on-site surgeon takes considerably longer. Since surgeons often have multiple cases to attend to while at the hospital, it is plausible that having a surgeon available all the time for teleconsultation would decrease time to management. However, Bracey et al. [33], finding of a substantial time increase between evaluation and final treatment after implementation of the AHTTP, argue for a management delay with teleconsultation, presumably due to a lengthy video consultation material preparation process. In light of this discrepancy, studies following similar methodologies should be conducted to clarify results.

## 3. Impact of Telemedicine for Remote Evaluation of Hand Injuries during the Current COVID-19 Pandemic

The initial months of the COVID-19 pandemic were characterized by the implementation of “stay at home” policies worldwide to mitigate viral spread and overwhelming health care systems. Consequently, this led to a shift in the injury patterns of traumatic injuries. In the case of hand injuries, there was a decreased incidence of sporting and motor injuries, while home-based accidents, such as those caused by knives and other tools, remained constant or increased [50,51,52,53,54,55]. Among these lesions, those occurring in the fingers were observed to be the most common [56]. These data highlight that the incidence of hand injuries can increase during lockdowns. Contrary to elective consultations and surgeries, the emergent nature of this type of injury requires implementing systems to diagnose, triage, and treat patients remotely and effectively in the setting of pandemic lockdowns. Understandably, telemedicine is harder to apply to emergent cases than elective ones, but it is crucial to leverage these tools to create systems that can be adapted to emergency responses.

The consequences of not having appropriate telemedicine tools for traumatic hand injuries can be severe, and are best represented by a case published by Svorai et al. [57]. In this article, the authors describe the case of a 26-year-old female that abstained from receiving medical treatment after a wrist laceration injury acquired at home due to fear of getting infected with COVID-19. The patient required a two-stage correction surgery to repair the complications of a complete tear of the flexor pollicis longus four months after the initial injury [57]. Importantly, this could have been prevented had she had access to a safe and efficient evaluation system. This case highlights the importance of endorsing the study of telemedicine for non-COVID conditions during the pandemic [57].

**Figure 1:**
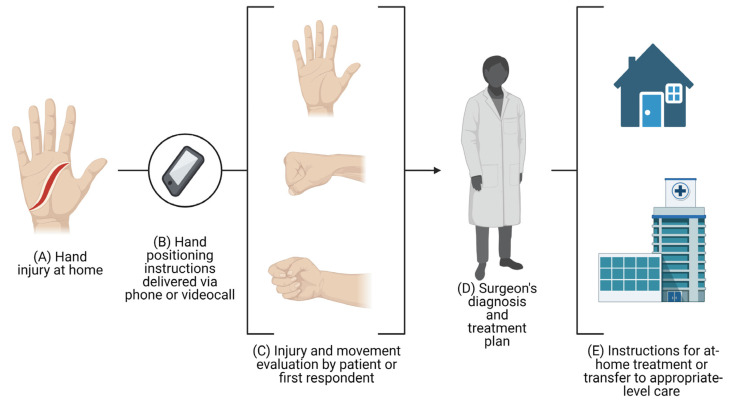
**At-home hand injury evaluation.** (**A**) With adequate standardization of hand injury photography, hand injuries could be evaluated on-site. (**B**) Patients could call the emergency services and receive specific hand positioning instructions to (**C**) perform their photo or video evaluation. (**D**) The files would then be sent to a surgeon to establish a diagnosis and treatment plan. (**E**) Lastly, the evaluating surgeon could provide instructions for at-home treatment or patient redirection to an appropriate-level health care facility. The figure was created using BioRender.com (www.biorender.com, accessed on 15 March 2023).

In the past, telemedicine was limited by cost and a lack of specialized equipment and trained staff [24]. With the advent of smartphones and electronic tablets, exchanging multimedia files has become an everyday practice. Although medical photography protocols and guidelines exist [58], no studies evaluate diagnosis or treatment differences between multimedia obtained by different users. In the included studies, multimedia files were obtained from users with varying levels of medical knowledge, resulting in non-standardized photographs and videos. Understanding how a user’s medical experience influences a surgeon’s diagnosis and treatment decisions could substantially impact how telemedicine is used, not only to evaluate traumatic hand injuries, but other types of acute injuries as well. Traumatic hand injury guidelines could implement recommendations on the correct physical examination steps that need to be followed for a surgeon to provide the most accurate diagnosis, allowing for the standardization of medical multimedia in this field of plastic surgery. This could eventually lead to immediate on-site injury evaluation using telemedicine interfaces such as video, photography, audio, or a mobile app (see Figure 1).

Telemedicine has been regarded as an asset to prevent infection spread by avoiding crowding the ED with patients that might be asymptomatic for SARS-CoV-2 infection [59,60]. The technologies presented in this study should be leveraged during the COVID-19 pandemic and future ones to improve telemedicine care for traumatic hand injury patients even after the public health crisis has passed.

Patient satisfaction is also currently regarded as a marker of quality care. Therefore, conducting implementation-oriented studies to explore the patient’s and the provider’s views on these new systems is crucial. Although patients are satisfied with the care received using telemedicine and virtual clinics, it has also been observed that most of them prefer to have in-person consultations given the opportunity outside a lockdown setting [61]. In the case of hand trauma, a retrospective study by Popova et al. [62] analyzed the patient experience after receiving follow-up visits using telemedicine or in-person appointments during the COVID-19 pandemic. The authors found that there is no difference in satisfaction between the two patient cohorts [62]. Therefore, satisfaction and willingness to participate in virtual clinics and telemedicine appointments may differ by the type of injury. Further research should be performed to identify the system characteristics that need to be modified to improve patient perception and increase satisfaction in virtual trauma hand injury clinics.

## 4. Legal Implications of Telemedicine in Traumatic Hand Injuries

The extreme relevance of the legal implications of the system proposed in this study and illustrated in Figure 1 cannot be underestimated. Such systems can undoubtedly lead to malpractice lawsuits for several reasons, including but not limited to delays in evaluation or treatment, incorrect diagnoses, improper risk discussion, and delayed referrals [63]. However, many of these reasons can be grouped as communication failure, as pointed out by Ernesäter, Engström, and Holmström [64]. Previous research on the topic shows that voice calls where the triage nursing staff used closed-ended questions had more malpractice lawsuits than those where more information was obtained from the patient [65]. This information highlights the need for a comprehensive virtual examination and a timely evaluation. To avoid severe and catastrophic events [66], telemedicine triage for traumatic hand injuries should leverage all the information exchange modalities of smartphones, such as photography, video, and voice.

It is unclear how a malpractice lawsuit would unfold after using a self-assessment telemedicine system for traumatic hand injury evaluation. The practicality of systems involving the exchange of different multimedia types is the recording and timestamping of every event in the patient’s evaluation, so that every party involved can access the proof easily. Additionally, the system should adhere to treatment guidelines strictly, such that an expert witness can agree to the approach provided after reviewing the patient’s case [67]. Although adherence to guidelines is a key element, other features are also relevant for a system whose legal safety relies on appropriate referral. As described by Wheeler et al. [68], for a system to be complete, it must include guidelines, documentation, training, standards, and a decision maker. Altogether, these factors compose a safe system. Based on these, the authors established that an appropriate referral (i.e., right time, place, and person) was possible with telephone triage and most often achieved by nursing staff and physicians [68]. There is therefore no reason a system involving not only voice, but several other interfaces could achieve these results as well. However, before turning such a system into reality, including these factors should be guaranteed to both patients and health care providers to create a safe legal environment.

## 5. Future Directions

The widespread use of mobile devices worldwide not only facilitates telemedicine delivery via video calls and regular voice calls, but also through mobile applications. This telemedicine interface is being explored [69], with varying results depending on the medical area where they are implemented [70,71,72]. Many current descriptions of mobile applications for trauma patients focus on triaging patients in disaster situations, as described in a recent review by Montano et al. [73,74]. Among the mobile apps that have been evaluated and described, there are some for prehospital evaluation and others that aid in patient stratification while in the ED. The latter includes that developed by Savatmongkorngul et al. [75], who developed a mobile version of the Emergency Severity Index, a triage tool used in the ED to stratify patients into five groups, from most to least urgent, based on their acuity and resource needs [76]. In this study, the authors found the highest inter-rater reliability rates between pairs of medical students and emergency physicians using the mobile app, while also showing that the subjects were more confident with the mobile version of the tool [75].

Another application is the “Major Trauma Triage tool” described by Freshwater and Crouch [77]. After noticing that emergency medicine service providers stored a version of a decision tree, the Trauma Unit Bypass tool, in their phones, the authors created a mobile version [77]. This tool combines anatomical injuries and physiological signs to guide hospital destination selection [77]. The developed application included the Trauma Unit Bypass tool, recommendations for hospital destination based on location, and collection and presentation of clinical handover information in an “ATMIST” format [77]. This format is a mnemonic composed of age, time of onset, medical complaint or injury, investigation, signs, and treatment [78]. The authors found that the app version of the tool performs as well as the paper version, and can aid prehospital emergency services in triage decisions related to major trauma [77].

Following the same objective of improving prehospital patient triage using mobile applications, Sutham et al. [79] developed an app to classify patients based on initial dispatch codes and criteria-based dispatch. In this case, the authors found the app evaluation to be faster and more effective than regular triage in non-trauma scenarios [79,80]. Furthermore, among other apps created for more specific purposes, FAST-ED helps prehospital emergency services to triage patients with cerebrovascular events to an appropriate hospital [81]. This app integrates a questionnaire assessing the patient’s status, a database of regional stroke centers, and GPS technology to calculate the transportation times to these facilities [81]. This last study is an ideal example of what a mobile application to evaluate traumatic hand injuries would need to include. Apart from instructions on evaluating the patient’s lesion, a database of trauma centers with the necessary infrastructure, material, and expertise would need to be included. Additionally, enabling GPS services with real-time traffic would be extremely valuable in cases of traumatic amputations to automatically identify the most suitable center and minimize ischemia time.

Such an application would be useful not only for prehospital health care providers, but also for patients. In a recent study, Gilbert et al. [82] developed the “ODISSEE” mobile application for patients, allowing them to self-evaluate and redirect them to the appropriate service provider. Among the suggestions given to patients are calling the emergency services, going to their preferred ED for specialized care, scheduling an at-home primary practice visit, or scheduling an in-person primary care visit for a different date [82]. When evaluated in a controlled setting, the application’s triage recommendation matched the expert’s advice in 85.6% of cases [83]. Notably, when the recommendations did not match, the application favored over-triaging [83]. Although further research is required before its implementation, this application proves that patient self-evaluation is possible and should be explored. Considering that signal instability, data usage, or bandwidth limitations in certain situations or geographical regions could render voice calls and video calls impossible, a mobile application for the self-evaluation of traumatic hand injuries with a store-and-send system could be a suitable answer for these issues. Clear explanations on how to evaluate the lesion using videos or photographs, shared with the emergency medical services in a store-and-forward system, could be included in such a mobile application. Immediate evaluation by a remote physician could then trigger the emergency medical services who, when considering the physicians’ advice, the available centers based on a trauma center database, and real-time GPS traffic evaluation, would transport the patient to an appropriate location.

## 6. Conclusions

The results in this study encourage and endorse telemedicine to provide medical attention in the setting of traumatic hand injuries and confirm that telemedicine triaging, particularly photography evaluation, is an adequate and feasible surrogate for face-to-face evaluation. Nonetheless, it is crucial to consider the components of a complete system to create a safe legal environment for all parties. Furthermore, exploring innovative telemedicine interfaces, such as mobile applications for emergency medicine service providers or patients, should be further explored as a way to improve hand trauma triage in settings were other telemedicine modalities could fail. 

## Figures and Tables

**Table 1:** **Summary of included studies.** Information on the authors, study design, devices used, type of multimedia file, and number of patients were collected. A summary of the authors’ methods and most relevant outcomes are also provided.

Author, Year, and Country	Study Design	Device	Photography or Video	Number of Patients Included in the Study	Summary of Methods	Outcomes
Buntic R.F. et al. [22], 1997, USA	Case report	Digital camera	Photography	1 patient	A photography of the injury and its radiography were emailed to consultant physicians	Successful reimplantation of the mutilated thumb
Hsieh C.H. et al. [23], 2004, Taiwan	Observational descriptive	Cell phone camera	Photography	45 patients (81 digits)	A photograph of the injury and a short trauma history were sent to the consultant surgeon, who triaged the patients into three groups. The patients were also triaged by three junior plastic surgery residents at a later time	15% of the cases resulted in treatment disagreement between final treatment and teleconsultation. A total of 20% of cases resulted in triaging disagreement. Remote diagnosis had a sensitivity of 79% and specificity of 71%, while remote recognition of bone exposure had a sensitivity of 76% and a specificity of 75%
Lam T.K. et al. [24], 2004, Australia	Observational descriptive	Cell phone camera	Photography	27 patients	Photos of the lesion(s) were taken by the resident in the ED and cases were discussed with the consultant surgeon, who established treatment before seeing the pictures	Treatment changed after photo inspection in four cases
Hsieh C.H. et al. [25], 2005, Taiwan	Observational descriptive	Cell phone camera	Photography	35 patients (60 digits)	A photography of the amputated portion and stump were sent to the consultant surgeon along with patient information and its radiograph. The images were evaluated by three other remote plastic surgeons.	The three remote surgeons correctly identified amputation location and status in 90% and 87% of cases, respectively, identified distal skin ecchymosis along the digital artery with 79% sensitivity and 90% specificity, and identified digital replantation potential with 90% sensitivity and 83% specificity
Diver A.J. et al. [26], 2008, United Kingdom	Observational descriptive	Digital camera	Photography	20 patients (17 with hand injuries)	A surgery resident assessed the patient and took pictures of the lesion, which were then taken to the attending surgeon along with the patient history to provide a preliminary management decision. After this, the attending surgeon examined the patients in person and final management was prescribed	High (95%) agreement by the attending surgeon with photography and description and high (95%) agreement between preliminary and final treatment. Five of the twenty patients (25%) could have been managed without attending the ED
Althubahati G. et al. [27], 2011, USA	Case series	Cell phone camera	Video	4 patients (1 patient with hand injury)	Consultations of patients with a requested urgent transfer were supplemented with videos of specific points in physical examination taken by the hand surgery fellow. Based on the described clinical picture and the video, the hand surgeon decided whether to accept or decline the transfer	Out of the four included cases that required transfer based on initial diagnosis, only one (25%) was considered for transfer after video evaluation by the attending surgeon
Waterman B.R. et al. [28], 2014, USA	Observational descriptive	Not specified	Photography	597 consults (197 hand injuries)	Using the AKO e-mail system, the on-site clinical team showed photos and a description of the case to an orthopedic surgeon, who decided on whether to evacuate the patient for tertiary care or treat him or her on-site	Teleconsultation prevented medical evacuation of 11 hand injury cases (out of 30 cases for which evacuation was initially requested)
Hara T. et al. [29], 2015, Japan	Observational descriptive	Cell phone camera	Photography	474 patients	The EMTs took photos of the injured fingers (following the hand surgeon’s instructions), which were sent to the investigators to assess the necessity for specialized treatment and redirect the ambulance to the most appropriate hospital	Acceptance to a hospital after three or fewer requests significantly increased (*p* = 0.039) after implementation of the Interactive Teletriage (from 79.2% to 86.4%)
Dehours E. et al. [30], 2016, France	Case series	Not specified	Photography	5 patients (1 finger injury)	A photo of an injured finger was taken by a civilian with limited training to the French Tele-Medical Assistance Service, who declined patient evacuation and advised for on-site treatment of the wound	Out of the five cases, there was only one evacuation
Paik A.M. et al. [31], 2017, USA	Observational descriptive	Tablet	Photography	42 patients (31 patients had hand injuries)	Patients with acute hand and facial wounds took pictures of their lesions, which were shown to a PSE, who then provided educational materials for the ED physician to make treatment and triage decisions. At the same time, patients were triaged, and a surgeon was consulted on-site, answering in person or by phone	Agreement rate between consultant and PSE was 90.5%. The mean response time for consultants was 48.3 min, while for PSE, it was 8.9 min, showing a statistically significant time reduction (*p* < 0.001)
Tripod M. et al. [32], 2018, USA	Cross-sectional	Tablet	Video and/or photography	202 patients (with isolated hand injuries)	The UAMS institutional trauma registry was queried for isolated hand injuries for the 2012–2015 period and subsequently divided into pre-AHTTP and post-AHTTP for transfer and costs assessments	In the pre-AHTTP group, 47.8% of patients were discharged home, while 52.2% were admitted or underwent surgery. In the post-AHTTP group, 31.8% patients were discharged home, while 68.2% were admitted or underwent surgery, resulting in a significant difference (*p* = 0.02). The direct cost of transportation for patients was also significantly lower in the post-AHTTP group (38.5% pre- vs. 21.4% post-, *p* < 0.0001)
Bracey J.W. et al. [33], 2019, USA	Cross-sectional	Tablet	Video and/or photography	331 patients, of which 298 had a telemedicine consultation (65% of these had videoconsultation)	The authors reviewed data on hand trauma from the first year of the AHTTP (2014) and compared it to the year prior (2013). Data collection focused on number of hand consultations, need for transfer, and time to disposition	After implementation of the AHTTP, transfers decreased from 73% in de pre-system period to 45% (*p* < 0.001). Time to disposition increased by 31 min on average (*p* < 0.001)
Westley S., Mistry R., and Dheansa B. [34], 2021, United Kingdom	Cross-sectional	Phone	Video or telephone using supporting photographs	126 patients evaluated in the virtual clinic; 99 patients evaluated in the face-to-face clinic	Trainees were asked to predict what treatment was required for patients in face-to-face visits (prior to COVID-19 lockdown) or virtual clinic	87% of patients evaluated virtually had an accurate assessment and all injured structures were correctly predicted, no patient had an unnecessary procedure—No significant difference in accuracy between video or telephone assessments (*p* = 0.88); 78% of patients in the face-to-face clinic had an accurate assessment, with no unnecessary procedures; no significant difference in overall accuracy between both clinics (*p* = 0.27)
Bracey J. et al. [35], 2021, USA	Cross-sectional	Tablet	Video and/or photography	331 patients, of which 298 had a telemedicine consultation (65% of these had videoconsultation)	The authors reviewed data on hand trauma from January 1 to December 31, 2014 (first year of the program). Data focused on type of telemedicine consultation, need for transfer, and type of transfer recommended (general orthopedic vs. hand surgeon)	Out of 298 telemedicine consultations, 195 (65%) used video and 103 (35%) used phone only (both groups had access to imaging studies); of the patients using video, 91 (47%) were transferred and 60 (58%) were managed locally; of the patients using phone only, 43 (42%) were transferred and 60 (58%) were managed locally; using video did not significantly affect the decision to transfer (*p* = 0.42)

AHTTP, Arkansas Hand Trauma Telemedicine Program; AKO, Army Knowledge Online; ED, Emergency Department; EMT, Emergency Medicine Technician; PSE, Plastic Surgery Educator; UAMS, University of Arkansas for Medical Sciences.

## Data Availability

The databases generated for drafting this manuscript can be solicited from the author upon reasonable request.
